# Phylogenomic analysis of cytochrome P450 multigene family and their differential expression analysis in *Solanum lycopersicum* L. suggested tissue specific promoters

**DOI:** 10.1186/s12864-019-5483-x

**Published:** 2019-02-07

**Authors:** A. P. Vasav, V. T. Barvkar

**Affiliations:** Department of Botany, Savitribai Phule Pune University, Pune, 411007 India

**Keywords:** Cytochrome P450, Phylogeny, Intron map, Genome-wide promoter analysis, Tissue specific promoter

## Abstract

**Background:**

Cytochrome P450 (P450) is a functionally diverse and multifamily class of enzymes which catalyses vast variety of biochemical reactions. P450 genes play regulatory role in growth, development and secondary metabolite biosynthesis. *Solanum lycopersicum* L. (Tomato) is an economically important crop plant and model system for various studies with massive genomic data. The comprehensive identification and characterization of P450 genes was lacking. Probing tomato genome for P450 identification would provide valuable information about the functions and evolution of the P450 gene family.

**Results:**

In the present study, we have identified 233 P450 genes from tomato genome along with conserved motifs. Through the phylogenetic analysis of *Solanum lycopersicum* P450 (*Sl*P450) protein sequences, they were classified into two major clades and nine clans further divided into 42 families. RT-qPCR analysis of selected six candidate genes were corroborated with digital expression profile. Out of 233 *Sl*P450 genes, 73 showed expression evidence in 19 tissues of tomato. Out of 22 intron gain/loss positions, two positions were conserved in tomato P450 genes supporting intron late theory of intron evolution in *Sl*P450 families. The comparison between tomato and other related plant P450s families showed that CYP728, CYP733, CYP80, CYP92, CYP736 and CYP749 families have been evolved in tomato and few higher plants whereas lost from *Arabidopsis*. The global promoter analysis of *Sl*P450 against all the protein coding genes, coupled with expression data, revealed statistical overrepresentation of few promoter motifs in *Sl*P450 genes which were highly expressed in specific tissue of tomato. Hence, these identified promoter motifs can be pursued further as tissue specific promoter that are driving expression of respective *Sl*P450.

**Conclusions:**

The phylogenetic analysis and expression profiles of tomato P450 gene family offers essential genomic resource for their functional characterization. This study allows comparison of *Sl*P450 gene family with other Solanaceae members which are also economically important and attempt to classify functionally important *Sl*P450 genes into groups and families. This report would enable researchers working on Tomato P450 to select appropriate candidate genes from huge repertoire of P450 genes depending on their phylogenetic class, tissue specific expression and promoter prevalence.

**Electronic supplementary material:**

The online version of this article (10.1186/s12864-019-5483-x) contains supplementary material, which is available to authorized users.

## Background

Cytochrome P450 (P450) belongs to a very divergent multigene family present in all living organisms. In angiosperms, approximately 300 genes are speculated per genome in 50 plant families [1]. The P450 monooxygenases are heme-thiolate enzymes, which catalyse broad range of chemical reactions like epoxidation, sulfoxidation, dehalogenation, dealkylation, C-C cleavage, ring extension, and reduction with the help of oxygen and NADPH [2]. They are involved in the oxidative metabolism of various endogenous and exogenous compounds like herbicides, pesticides and xenobiotics [3, 4]. The P450 proteins, present in plants are membrane bound and difficult to characterize [5]. The molecular mass of P450 from plant origin ranges from 45 to 62 kDa with an average molecular mass of 55 kDa. They possess four conserved key domains namely heme binding domain, I-helix, K-helix and PERF/W domain [6]. Heme-binding signature motif has 10 conserved residues among which cysteine is highly conserved. This heme-iron motif has a binding site for oxygen and various compounds involved in drug metabolism [7]. The P450 gene family is third largest gene family present in *Arabidopsis*. Most of the P450 studied in plants are localized in the endoplasmic reticulum, chloroplast or mitochondria and other secretary pathways [8]. They are involved in many biosynthetic pathways such as alkaloids, flavonoids, lignans, isoprenoids, phenolics, antioxidants and phenylpropanoid [8–10]. The P450 genes are crucial in metabolism and tolerance to allelochemicals in plants as well as in animals [11]. The gene families CYP90, CYP724 and CYP734 are involved in biosynthesis of steroidal saponins and glycoalkaloids. Different types of glycoalkaloids present in all members of Solanaceae family are vital compounds but toxic to other living organisms [12]. The P450 proteins are involved in the biosynthesis of aglycones from cholesterol by oxygenation, transamination and cyclization at different carbon positions. The P450 mediated derivatization of glycoalkaloids made them less toxic and during course of domestication solanaceae members with less amount of toxic glycoalkaloids have been selected [13].

Availability of whole genome sequences of large number of plant species allowed the genome wide identification of P450 multigene family in different plant species, namely soybean (*Glycin max*) [14], mulberry (*Morus notabilis*) [15], flax (*Linum usitatissimum*) [16] and tobacco (*Nicotiana tabacum*) [17]. The draft genome sequence of tomato (*Solanum lycopersicum*) was made publicly available in 2012 which provides an opportunity for genome-wide study of tomato specific gene families [18]. Tomato (*Solanum lycopersicum* L.) is an economically important crop and routinely used model plant for fruit ripening, plant-pathogen interaction and molecular genetics mapping [19]. However, very few P450 genes have been reported and functionally annotated from Tomato. Moreover, no comprehensive genome-wide study of these genes has been reported until date. Therefore. in this study, we attempt to classify functionally important P450 genes into groups and families according to standard P450 nomenclature committee [20, 21]. Understanding the molecular evolution, differential expression in different tissue types as well as intron and promoter analysis of *Sl*P450 genes will pave the way for functional characterization of important candidate genes.

## Methods

### Identification of P450 genes from the tomato genome

The *Arabidopsis thaliana* P450 genes were downloaded from ‘The Cytochrome P450 homepage’ reported by D. R. Nelson (http://drnelson.uthsc.edu/CytochromeP450.html) [21]. These 254 *Arabidopsis* P450 sequences were treated as a query to perform BlastP search with the E-value ≤1e^− 40^ against tomato (*Solanum lycopersicum*) genome (ITAG2.3) available at Phytozome database V10 (www.phytozome.net) [18]. Furthermore, a manual analysis of putative *Solanum lycopersicum* P450 (*Sl*P450) sequences was conducted for the complete ORF and truncation. The analysis consist of non-redundant and full-length *Sl*P450 genes. Universal names for *Sl*P450 genes were assigned according to the standard system of P450 nomenclature committee [20, 21].

### Multiple sequence alignment, phylogenetic tree construction and conserved motif analysis

The 48 P450 protein sequences from other plants such as *Arabidopsis thaliana* (40), *Populas trichocarpa* (1) and *Solanum tuberosum* (7) along with 233 *Sl*P450s from *Solanum lycopersicum* were considered to construct the phylogenetic tree. The accession numbers are provided in Additional file 1. Multiple sequence alignment of these P450 genes was carried out with Muscle algorithm [22] using default parameters present in MEGAX software [23]. The phylogenetic tree was constructed using Neighbour-joining (NJ) [24] and maximum likelihood (ML) algorithm. The Dayhoff substitution matrix (PAM250) along with the bootstrapping (1000 replicates) was employed for NJ analysis. The unrooted maximum likelihood phylogenetic tree and evolutionary analyses were carried out using IQ-TREE web server (http://iqtree.cibiv.univie.ac.at/) [25]. The best-fit model was selected from 168 amino acid substitution models using modelfinder tool [26]. The modelfinder reported LG + F + I + G4 as best-fit model according to bayesian information criterion (BIC score 420,547.05). The ML tree was built with 1000 ultrafast bootstrap [27] replications and the final tree with highest log likelihood (− 208,278.21) was considered for phylogeny inferences. For conserved domain identification, multiple sequence alignment of *Sl*P450 protein sequence were carried out using Clustal X program using default parameters [28]. The alignment file was submitted to Web Logo generator software for generating the logo of conserved domains available at (http://weblogo.berkeley.edu/) [29].

### Intron map and their organization

Intron map of tomato P450 genes was drawn by using previously described methods suggested by Barvkar et.al. and Paquette et.al. [30, 31]. The intron-exon boundaries, introns phases and their position in protein sequences were considered for the same. Introns present in genomic sequences, were mapped on protein sequences and serially numbered. Introns can have three intron phases: intron phase 0, 1 and 2. Introns with the identical positions in one codon along with similar intron phase are termed as ‘conserved intron’. The intron map was constructed by considering 145 (62.23%), *Sl*P450 genes sequences with one and two introns.

### Promoter analysis of *Sl*P450 genes and identification of tissue specific promoters

The promoter analysis of tomato P450 genes helps to identify over-represented motifs regulating gene expression. We used previously characterized motifs from PLACE [32] and plant CARE databases [33] to obtain regulatory motifs which are over-represented in a group of genes. The consensus motifs from these databases were used since it has high coverage of previously characterized plant motifs (total 946 plant motifs). The complete *Solanum lycopersicum* genome was downloaded from Phytozome database. Moreover, the bed file with genomic coordinates was used to extract 2 kb upstream sequence of all the protein coding genes using bedtools suite with getfasta option [34]. The promoter motifs for all protein-coding genes were identified using perl script generously shared by Dr. Angelica Lindlöf [35]. The presence of core promoter sequence can occur randomly because of the short length. Hence, we excluded random occurrence probability of any promoter motif in *Sl*P450 upstream sequence. To calculate non-random occurrence probability, the presence or absence of individual promoter motif in two groups was compared statistically. The first group included *Sl*P450 genes highly expressed in specific tissue types (Leaf, buds, peel, petals, roots, seeds) and the second group contains all the protein coding genes. The statistical one-sample test for binomial proportions was applied at significant *p*-value (≤ 0.05). We used fragments per kilobase of transcript per million mapped reads (FPKM) values from RNA sequencing of various tissue types to understand the relationship between promoter occurrence and actual gene expression of individual *Sl*P450 gene. Furthermore, a comparison was carried between previously mentioned two groups. The motifs which are statistically significantly overrepresented were assigned as tissue specific promoter motif that are driving expression of selected *Sl*P450 genes.

### Digital expression analysis of *Sl*P450 genes

The digital expression analysis was performed to gain an insight of the role of the identified *Sl*P450 in the various tissues. We used publicly available RNA-sequencing data from Dr. Asaph Aharoni lab (https://www.weizmann.ac.il/plants/aharoni/sites/plants.aharoni/files/uploads/tomato_rnaseq_data_19_tissues.xlsx) in order to decipher expression of *Sl*P450 in 19 different tissues namely leaf, root, floral buds, petals and peel, flesh, seeds of immature green, mature green, breaker, orange and red fruits respectively. Available RNA-sequencing data were normalized with FPKM method. Digital expression profile of *Sl*P450 genes in the form of heat map was constructed using ClustVis software (http://biit.cs.ut.ee/clustvis/) with default parameters [36].

### Plant material

The *Solanum lycoperscium* L. cv MicroTom (TGRC accession number: LA3911) seeds were generously provided by Prof. Asaph Aharoni (Department of Plant Sciences, Weizmann Institute of Science, Israel) which were obtained from Tomato Genetics Resource Center (http://tgrc.ucdavis.edu). The Tomato plants were grown in the poly house and maintained at controlled conditions of temperature (25 °C) and humidity (54%). On maturation of plants, root (R), stem (S), leaves (L), flower (F), green fruit (GF), mature green fruit (MGF) tissues were harvested. The tissues were frozen in liquid nitrogen and stored at − 80 °C until further use.

### Real-time quantitative PCR (RT-qPCR) analysis

To confirm the digital expression analysis of *Sl*P450s, we have selected six genes i.e. *SlCYP51G1*, *SlCYP90A5*, *SlCYP77A20*, *SlCYP71AX11*, *SlCYP74C3* and *SlCYP733A* depending on their higher expression in various tissues. Total RNA from root, stem, leaves, flower, green fruit, and mature green fruit tissues were extracted using trizol reagent (Invitrogen, USA) [37] as per the manufactures protocol. Total RNA was quantified with NanoDrop (ND-1000 spectrophotometer, Wilmington, USA) and then treated with RNase-free DNaseI (Promega, USA) to remove DNA contamination. Total 2 μg of RNA was reverse transcribed into cDNA by using AMV reverse transcriptase (Applied biosystems, USA) [38]. The cDNA synthesized from different tissues were used for RT-qPCR analysis. Primers for RT-qPCR were designed using Primer 3 software available at (http://bioinfo.ut.ee/primer3-0.4.0/). The primer sequences are available in the Additional file 2. RT-qPCR analysis was performed in the Realflex2 Master cycler (Eppendorf, Germany). We used 5 μl of 2x SYBR green master mix (Roche, USA), sterile milliQ water, 10pM forward and reverse primer and 1.5 μl (1:3 diluted) cDNA for RT-qPCR analysis. Thermal profile used for RT-qPCR analysis were as follows: initial denaturation at 95 °C:5 min followed by 95 °C:15 s, 60 °C:30 s, 72 °C:30 s for 40 cycles. After amplification, melting curve analysis was conducted at 60–95 °C ramps with 0.5 °C increment per cycle to check the primer specificity. Elongation factor one alpha (*EF1α* NCBI Acc No. NM_001247106) gene was used as housekeeping/internal control after verifying the uniform expression in all the studied tissues of tomato. Relative expression profile of selected six candidate genes *SlCYP51G1*, *SlCYP90A5*, *SlCYP77A20*, *SlCYP71AX11*, *SlCYP74C3*, *SlCYP733A* were determined by using 2(−Delta Delta C(T)) Method as described by Livak et al. [39]. Each gene had a PCR efficiency and R^2^ value between 0.9–1.00 along with single melting curves. The experiment was repeated with three biological and two technical replicates for each gene.

## Results

### Annotation and classification of tomato P450 multigene family

**Fig. 1:**
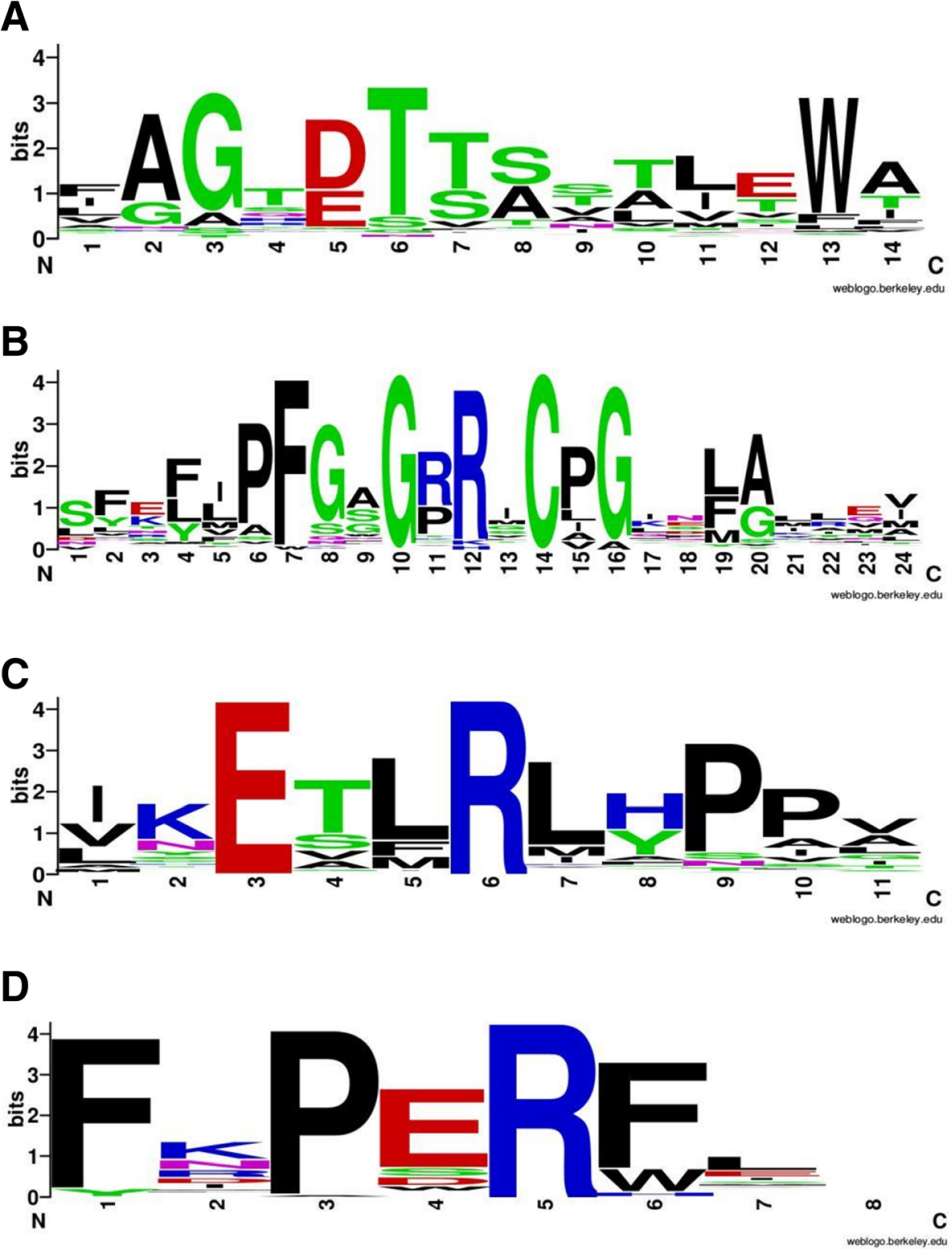
Conserved motifs/sequence logos of the predicted tomato P450 proteins: Web Logos of conserved motifs in P450 from tomato, *A. thaliana*, *P. trichocarpa* and *S. tuberosum* P450 sequences. Letter size in the logos is proportional to the degree of conservation. **a** AGxDT (I- helix), (**b**) Heme binding motif, (**c**) KETLR (K-helix), (**d**) PERF/W motif respectively

A total of 300 tomato P450 genes were identified from tomato genome which includes full length, pseudo genes and truncated genes. Moreover, 233 putative non redundant full length P450 gene sequences were identified using BlastP search. All four conserved key motifs i.e. heme binding domain, I-helix, K-helix and PERF/W motif were part of it. These sequences possess complete ORF and amino acid length that varies from 450 to 600 residues with an average of 505 amino acids. The average percent identity of 233 *Sl*P450 proteins was 25.87 and ranges from 95.7 to 13.7. The isoforms of 94B (*Sl*CYP94B18 and *Sl*CYP94B20) showed maximum percent identity, whereas pair *Sl*CYP74C4 and *Sl*CYP701A30 exhibited minimum percent identity (Additional file 1). Four conserved motifs are shown in the Fig. 1. These are similar as previously described by Bak et al. [5].

### Phylogenetic analysis of the tomato P450 multigene family

**Fig. 2:**
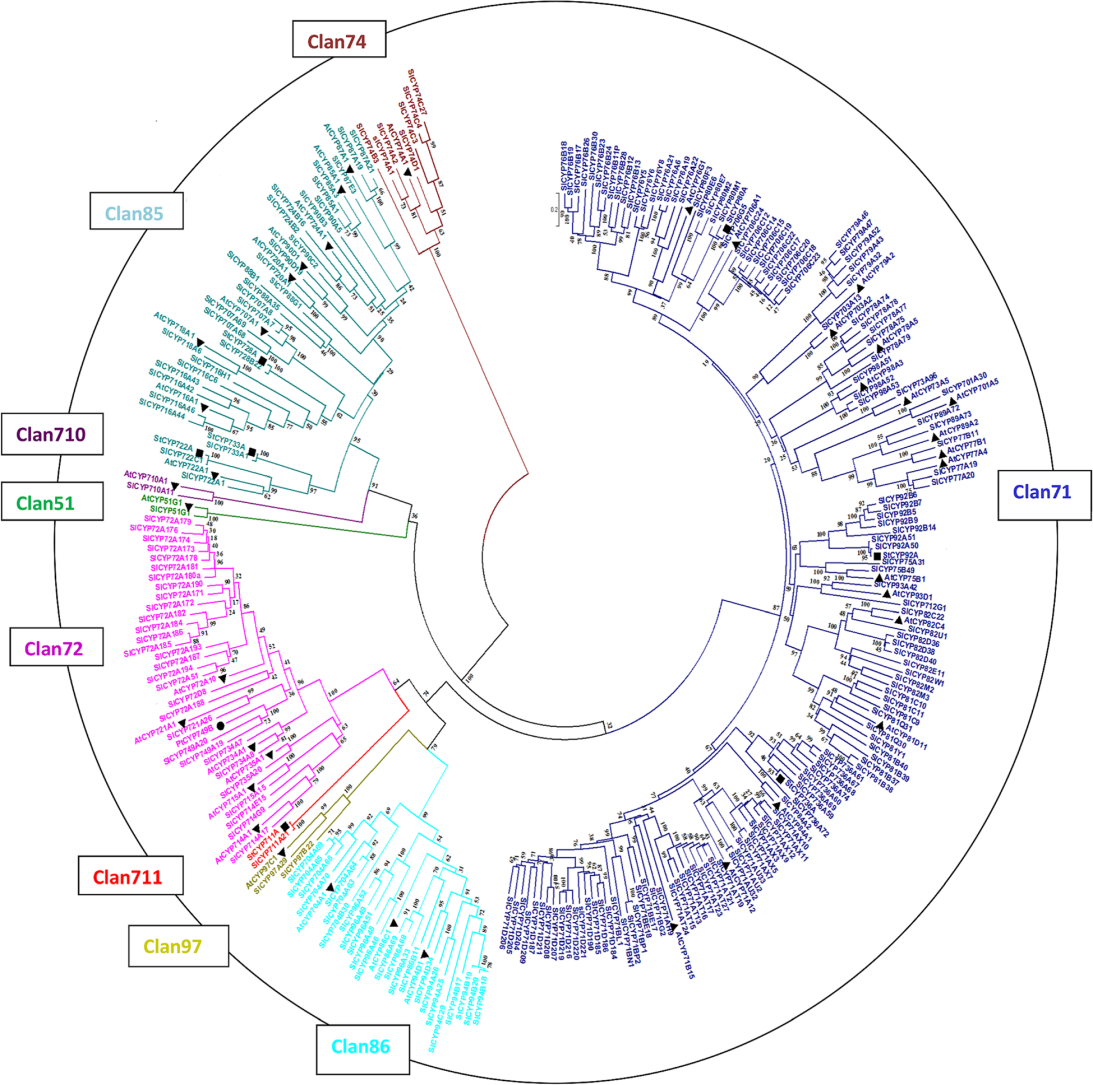
Phylogenetic tree of the tomato P450 genes: Phylogenetic tree is constructed by using NJ algorithms with 1000 bootstrap replicates. Different clans are represented by different colours. The abbreviations used for different plant P450 protein sequences are as follows: Sl- *Solanum lycopersicum*, ▲(At - *Arabidopsis thaliana*), ■(St -*Solanum tuberosum*), ●(Pt - *Populus trichocarpa*)

The phylogenetic tree of *Sl*P450 proteins divided into two major clades: A-type and non-A type. These two clades are further clustered into nine clan i.e. clan71, clan51, clan710, clan85, clan711, clan86, clan97, clan72, and clan74 [Fig. 2]. The tree topology of NJ and ML tree [Additional file 3] is similar therefore, it indicate the robustness of phylogenetic tree and clustering of *Sl*P450 genes into families and clans. Phylogenetic analysis revealed that clan51, clan710, clan711 and clan74 are single family clans; remaining five clans contain multiple families of *Sl*P450 genes [40, 41]. Overall *Sl*P450 genes are classified into 42 families. The 137 (59%) *Sl*P450 genes are designated as A-type and can further be divided into 21 families while 96 (41%) *Sl*P450 genes are assigned as non-A type and can be classified into 21 families. In tomato genome clan71 comprises more than 50% genes. The CYP71 family is largest A-type family which contains 43 (31.61%) genes divided into 10 subfamilies i.e. CYP71D, CYP71AH, CYP71AT, CYP71AU, CYP71AX, CYP71BG, CYP71BE, CYP71BL, CYP71BN and CYP71BP. The clan 72 has eight subfamilies whereas CYP72 is the largest non-A family which contains 20 (20.83%) genes. It is further divided into two subfamilies namely CYP72A and CYP72D [5]. During the course of evolution CYP728, CYP733, CYP80, CYP92, CYP736 and CYP749 families were evolved in tomato genome and lost from *Arabidopsis* genome. *Sl*CYP51, *Sl*CYP710 and *Sl*CYP85 clans cluster together in the phylogenetic tree indicating paralogous origin. *Sl*CYP74 clan has four subfamilies and act as outgroup in the phylogenetic tree since it is an atypical plant P450 clan which lacks the monooxygenase activity. The clan 97 and 86 appears to share common ancestral genes and therefore they are clustered together in the phylogenetic tree.

### Intron gain and loss events to investigate evolution of P450 multigene family

**Fig. 3:**
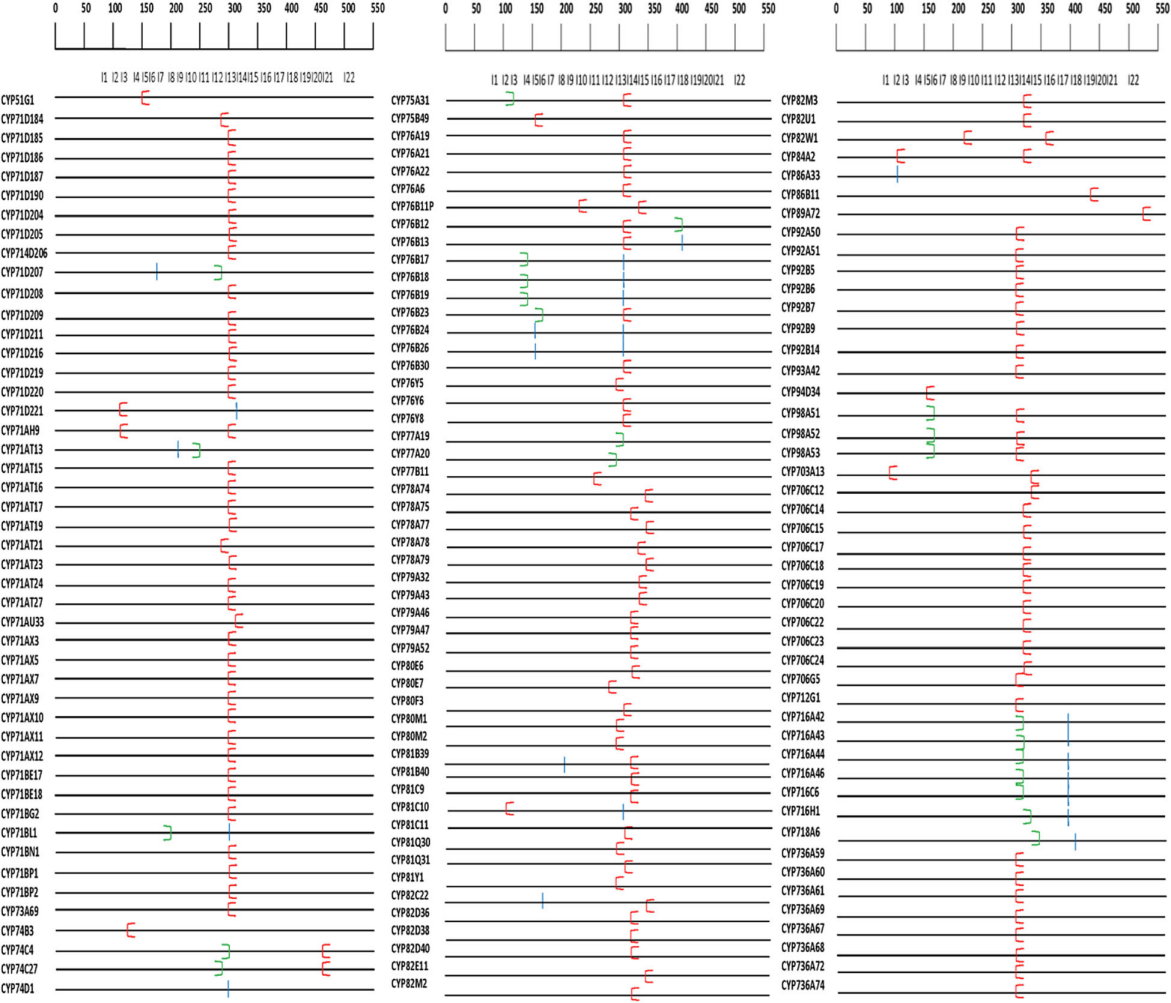
Intron distribution of 145 tomato P450 genes in intron map: Number on top of intron map indicates the independent intron insertions occurred in each gene. Intron positions are mapped on their amino acid sequences. Three intron phases present in genes indicated by different colour and symbols: [- intron phase 1,] - intron phase 2 and |- intron phase zero respectively

Understanding gain and loss of the intron reflects the evolution of gene family. In the present study, we analysed the intron number and phases. The identified *Sl*P450 genes have minimum zero and maximum 14 introns. Out of 233 *Sl*P450 genes, 23 (9.87%) genes have no intron, 108 (46.35%) genes have one intron, 37 (15.87%) genes have two introns, four genes (1.71%) have three introns, 30 genes (12.87) have four introns and 31 genes (31.30%) contain five/ more than five introns. The intron map of P450 gene sequences was constructed by considering 145 genes that had one and two introns (comprising 62.23% of the total genes). The data used to construct the intron map and distribution graph are provided in Additional file 4. A total of 22 independent intron insertion events were occurred in *Sl*P450 genes [Fig. 3]. If intron position in a particular sequence was within 40–45 amino acids of its mean recorded position across the sequences, it was considered as conserved [30]. Introns number I13 and I14 are conserved in intron map. Intron map analysis revealed that most of the gene families contain conserved intron I13 (56.55%) and I14 (17.93%). These two introns are recent introns amongst identified 22 introns. Both conserved introns are present in gene families belonging to clan71. Families with conserved intron I13; lack conserved intron I14 and vice versa. For example, CYP84 family gene has conserved intron I13 whereas it lost the conserved intron I14 and contains additional intron at I2 insertion site. It was observed that I13 intron has evolved during the course of evolution and I14 intron was lost from *Sl*P450 genes (Fig. 3). In the intron map, 122 (84.13%) genes have conserved intron I13 and the remaining genes have conserved intron I14 at intron insertion site. Out of 145 genes, 106 genes have intron phase one and 39 genes have intron phase zero and two. It was observed that gene families from same phylogenetic group have similar intron numbers and organization. The *Sl*P450 genes belonging to non-A type families lack conserved introns but have introns at different intron insertion sites. For example, *SlCYP51* from clan51 lost both the conserved introns, gained I5 intron and created separate family. The *SlCYP718A6* and family *Sl*CYP716 genes that belongs to clan85 also lost both the conserved introns, gained I18 intron and diverged. Both the conserved introns were in the same intron phase and only appear in A-type P450 clan. This suggests the recent diversification of A-type P450 genes from common ancestral gene and neofunctionalization during the course of evolution [30].

### In silico analysis of tomato P450 gene promoters

Promoter motifs play crucial role in execution of the biological functions of the genes. The comparisons were carried out between group of *Sl*P450 which were highly expressed in different tissue types with all the protein coding genes in tomato. The list of over-represented motifs obtained from promoter analysis of 233 tomato P450 genes are enlisted in Additional file 5. Among 233 *Sl*CYPs, 73 (31.33%) genes which had digital expression evidence were considered for further promoter analysis. Specific over-represented promoter motifs from selected tomato tissues specific P450 gene are summarised in Table 1. with their probable biological function.

**Table 1:** Tissue specific *SlP450* having over-represented promoter motifs along with their probable biological role

Sr. No	Over represented Motif name	Tomato Tissue Type	Biological function	Solyc Id	Universal name
1	AC motif and MYB1LEPR motif	Leaf specific P450 genes	These motifs are present in bean phenylalanine ammonia-lyase (PAL) gene and together play crucial role to co-ordinate regulation of phenylpropanoide metabolism [41, 42, 43]	Solyc04g071800 Solyc11g006590 Solyc04g011690	*SlCYP92B7* *SlCYP71AT7* *SlCYP736A*
3	AGL motif	Root specific P450 genes	*Arabidopsis* AGL19 and AGL18 promoter motif showed specific expression in root meristem and central cylinder cell in mature root and also in petals and siliques [44, 45, 46, 47].	Solyc12g042480 Solyc02g084570 Solyc03g120060 Solyc10g017510 Solyc05g047680	*SlCYP736A4* *SlCYP84A2* *SlCYP734A8* *SlCYP71BE8* *SlCYP78A77*
4	AT-box motif	Buds specific P450 genes	AT rich binding sequence characterized from promoter of tomato rbcs-3A gene. This motif meditate regulation of light harvesting gene complex [48, 49, 50].	Solyc04g078900 Solyc12g006460 Solyc07g043460	*SlCYP707A8* *SlCYP88B1* *SlCYP72A18*
5	Auxin responsive element	Root specific P450 genes	Soybean *GH3* gene has three auxin responsive element which are important in auxin mediated gene expression [51].	Solyc12g042480 Solyc02g084570 Solyc03g120060 Solyc10g017510 Solyc05g047680	*SlCYP736A4* *SlCYP84A2* *SlCYP734A8* *SlCYP71BE8* *SlCYP78A77*
6	HSE heat shock element	Root specific P450 genes	HSE are present in the heat shock proteins of *Apx1* gene and involved in oxidative stress defense. *Arabidopsis APX1* gene showed induced expression under oxidative stress [52, 53].	Solyc12g042480 Solyc02g084570 Solyc03g120060 Solyc10g017510 Solyc05g047680	*SlCYP736A4* *SlCYP84A2* *SlCYP734A8* *SlCYP71BE8* *SlCYP78A77*
7	TCP transcription factor	Petal specific P450 genes	TCP transcription factor involved in growth, development and defense mechanism also induces biosynthesis of Brassinosteroid (BR), Jasmonic acid (JA) and flavonoids might be involved in regulation of floral tissues developing genes in tomato plant. In *Arabidopsis* TCP14 and TCP15 motifs are involved in regulation of floral tissues and leaf blade development [54, 55, 56].	Solyc04g050620 Solyc07g062500 Solyc06g051750 Solyc08g079280 Solyc04g051190 Solyc08g080380 Solyc02g080330 Solyc10g080870 Solyc09g059240 Solyc11g065770	*SlCYP736A1* *SlCYP72A14* *SlCYP90A5* *SlCYP706C2* *SlCYP97A29* *SlCYP80E6* *SlCYP77B11* *SlCYP96A48* *SlCYP82U1* *SlCYP94A25*

### Digital expression profiling of tomato P450 genes

**Fig. 4:**
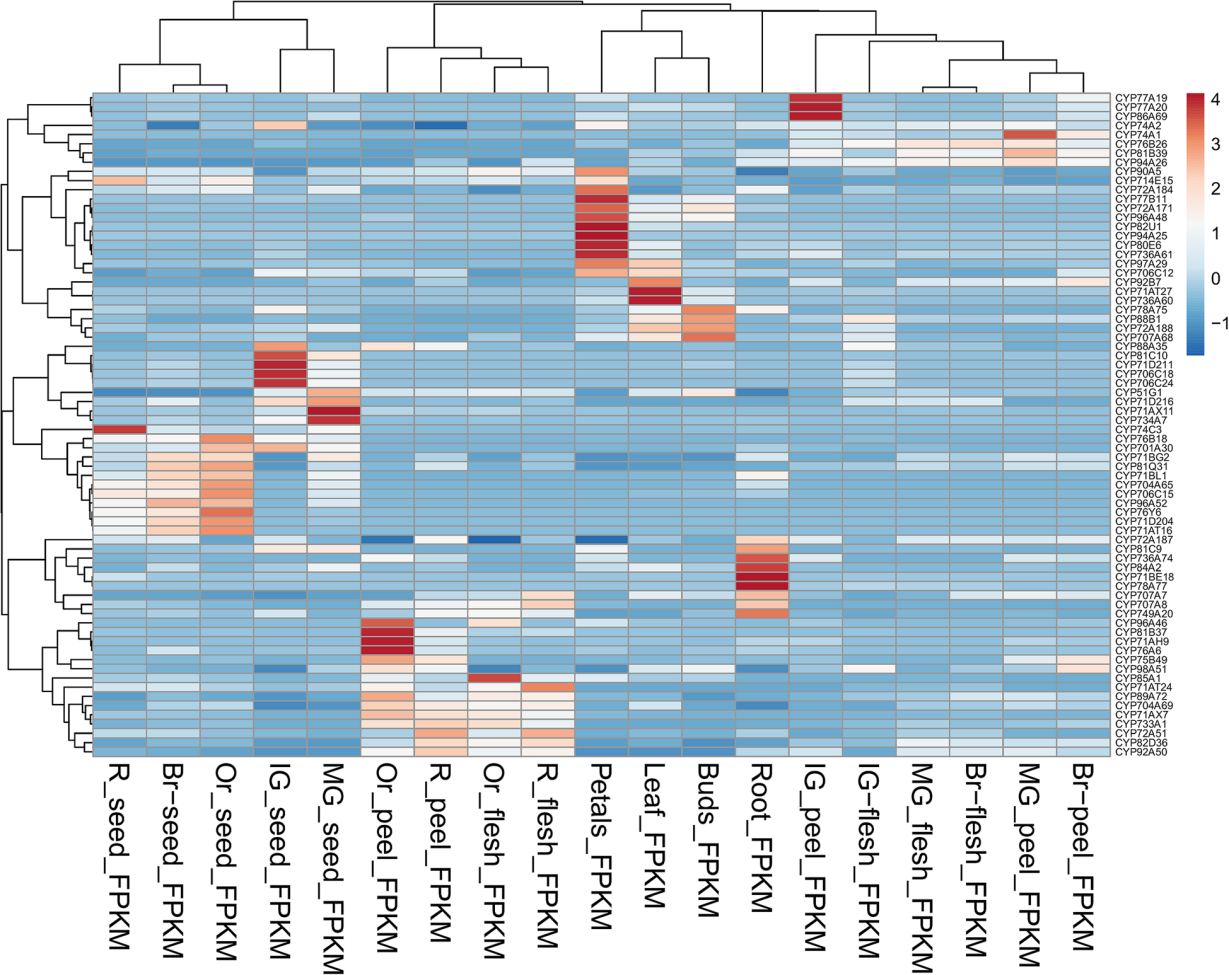
Expression profile of the 144 tomato P450 genes in the different tissue types using RNA sequencing data: The FPKM normalized values from RNA sequencing data of different tissues of the tomato was used to construct the heat map using ClustVis web server with default parameters. Colour scale is correlated with the expression of the genes: light blue colour- low expression and red colour- high expression. Abbreviations used for the tissues of tomato are as follows: R- red fruit, Br-breaker red fruit, Or-orange fruit, IG- immature green fruit, MG-mature green fruit

**Fig. 5:**
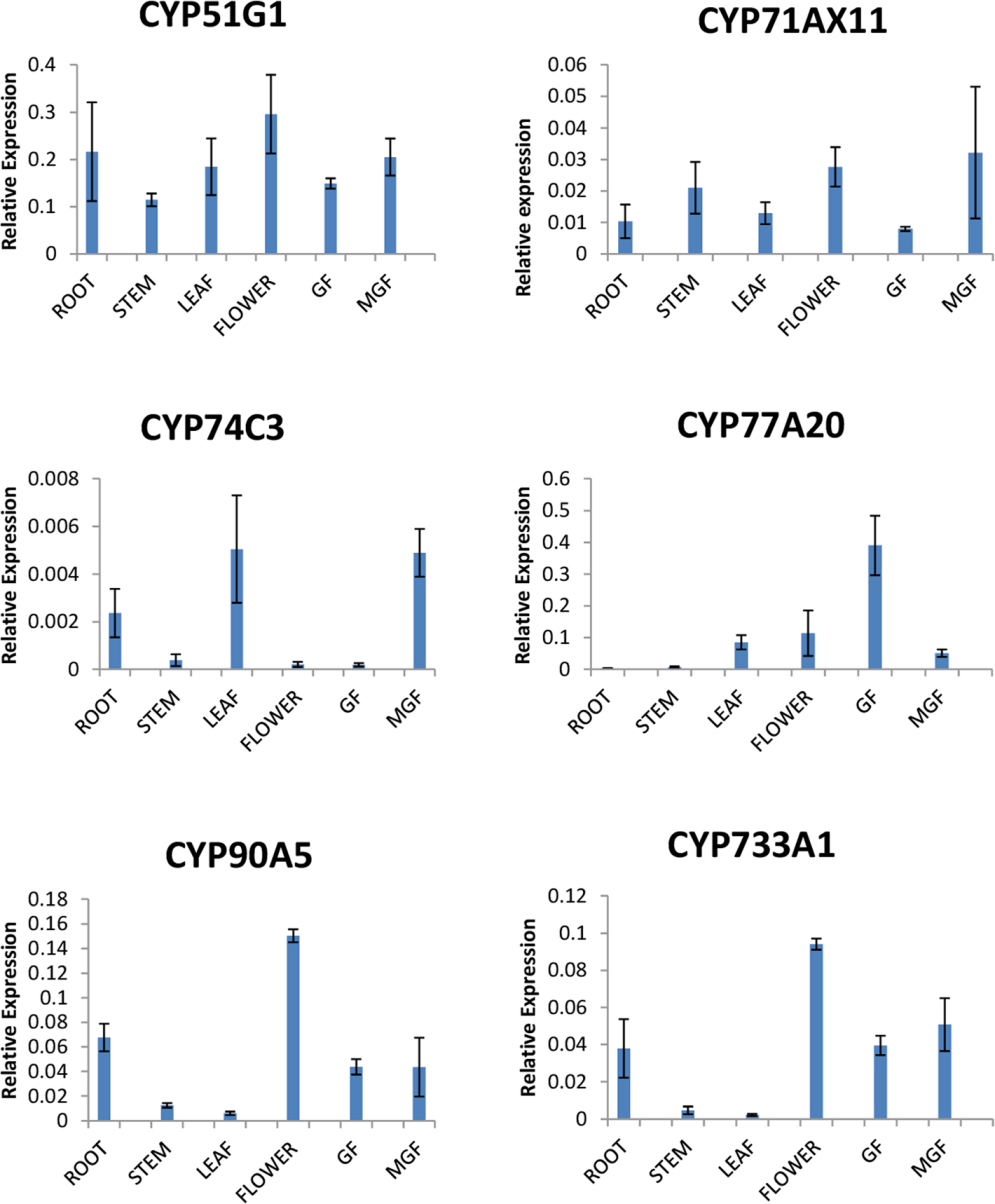
Expression of the selected candidate P450 genes in different tissue types: X-axis represents the different tissue types and y-axis denotes the relative expression values for the different genes. Bar indicate the mean value and Error bar indicates the standard deviation of the three biological replicates. Abbreviations used in graph for various tissues are as follows: GF- immature green fruit, MGF- mature green fruit

The FPKM normalized expression values were used to construct digital expression profile heat map [Fig. 4]. Among 233 *Sl*P450 genes, 73 (31.33%) genes were differentially expressed in different tissues. The developing seeds from different fruits ripening stages show large proportion (72.60%) of highly expressed P450 genes whereas least number of genes are expressed in buds (5.47%). Phylogenetic family specific expression of P450 genes varies from 2.38 to 930.98 FPKM (Additional file 6). Moreover, the digital expression was validated by RT-qPCR analysis of six candidate *Sl*P450genes that represented both single gene family and multigene family clades of tomato P450. These selected P450 were analysed for their relative transcript abundance and are graphically represented in Fig. 5. The *SlCYP51G1* exhibited 0.29 fold higher relative transcript abundance in flower. The *SlCYP77A20* and *SlCYP90A5* had 0.39 and 0.15 fold relative transcript upregulation in green fruit and flower, respectively which were corroborated with RNA sequencing data. The *SlCYP71AX11* showed 0.031 fold expression in mature green fruit. *SlCYP74C3* and *SlCYP733A1* genes had 0.005 and 0.094 fold relative transcript abundance in leaf and flower. The RT-qPCR analysis results were correlated with RNA sequencing data.

## Discussion

Cytochrome P450 genes are involved in catalysis of variety of reactions which include growth, development and secondary metabolite biosynthetic pathways. In present study we identified 233 P450 genes from tomato which are comparable with genes identified in *Arabidopsis thaliana* (245) [5] but more than mulberry (176) [15]. All identified tomato P450 genes contain four P450 signature conserved domains. The orthologs comparison of tomato P450 gene families with plant species such as *Arabidopsis*, *Medicago*, poplar, flax, moss, rice and soybean revealed the evolution of P450 gene family (Additional file 7). These results demonstrated that CYP702 and CYP708 families are present in *Arabidopsis* and absent from other analysed plants. This may be attributed to biosynthesis of triterpenoid derivatives that are Brassicaceae specific [57]. The *CYP749A20* gene was up-regulated in red and orange fruit with unknown function in tomato. However, its orthologue from *Arabidopsis* is absent. During the course of evolution, CYP749 family is evolved only in Asteroids, Rosides and Ranunculales members [5]. Tomato CYP78 family members have only CYP78A subfamily, interestingly genes from this family are involved in flower development and meristem specific function in *Arabidopsis* [5]. The *Sl*CYP78A sub-family genes, *SlCYP78A75* and *SlCYP78A77* were respectively up-regulated in flower buds and root. In addition, the *SlCYP78A77* also contains root specific promoter motifs i.e. auxin responsive element and HSE (heat shock element). These motifs are consequently involved in auxin mediated gene expression and combating oxidative stress in other plants [51, 52, 53]. The *Sl*CYP81 family has 10 genes distributed in four sub families which belong to clan71. The *Sl*CYP81B and *Sl*CYP81C subfamily genes were up-regulated during different stages of the tomato fruit development. It is demonstrated in *Arabidopsis* that CYP81D, CYP81F, CYP81H and CYP81G subfamily genes play important role in disease resistance [57, 58]. The *Sl*CYP81B and *Sl*CYP81C might be involved in tomato fruit development as well as protection from different diseases since they are highly expressed in these tissue types [41]. The CYP80 family is present in tomato, poplar and grape. It supposedly involved in phenolic coupling during alkaloid biosynthesis [59]. The *SlCYP80E6* gene found to be up-regulated in petals and it contain overrepresented TCP transcription factor which was a petal specific motif. In *Arabidopsis,* TCP transcription factor is involved in floral organs development and biosynthesis of different phytohormones [54, 55, 56]. Hence, *SlCYP80E6* is a potential candidate to study the floral development. The expression data suggests that *SlCYP84A2* gene was up-regulated in root and has root specific overrepresented AGL promoter motif. In *Arabidopsis, CYP84A1* gene is involved in the lignin biosynthesis. The functional analysis of this gene affects the lignification and vascular development [5]. Expression and promoter data from tomato suggests that *SlCYP84A2* gene might be involved in vascular development of the root.

Phylogenetic tree topology of tomato and *Arabidopsis* P450 revealed similar clustering that indicates conserved nature of P450 multigene family across the various plant species [20]. The single family clans contain low copy genes with essential function in all the plants. They restrict themselves from gene duplication due to strong purifying/negative selection process [60]. The CYP51 is ancient and conserved clan with single copy in all the phyla studied so far. The *SlCYP51G1* showed 82% identity with *AtCYP51G1* and it is involved in sterol metabolism [60]. RT-qPCR expression data has showed that *SlCYP51G1* is constitutively expressed in all selected tissues of tomato and has sterol demethylase activity required for the maintenance of membrane integrity [61]. The CYP71 family genes evolve through the gene duplication and seems to be recent in the evolutionary history [60, 61]. Tomato CYP71 family genes have average 30% sequence identity with *Arabidopsis* CYP71 family. The expression data of *SlCYP71AX* and *SlCYP77A20* from to clan71 showed that these two genes were up-regulated in green and mature green fruit of tomato which is in accordance with the transcriptome data. These two genes would be good candidates for the study of secondary metabolite synthesis in tomato fruit [62, 63].

The CYP74 family is an atypical plant P450 family and thought to be involved in catalysis of already oxygenated polyunsaturated C18 fatty acid hydroperoxide into other oxylipins [5]. The RT-qPCR data exhibited upregulation of *SlCYP74C3* gene in mature green tomato fruits and hence it could be a potential candidate gene to study oxylipin biosynthesis in tomato fruit. The *SlCYP90A5* gene was up-regulated in tomato flower and showed less expression in leaf which correlate with transcriptome data. The *AtCYP90A1* is involved in brassinosteroid metabolism and shows less expression in expanding leaf [58, 5] whereas tomato orthologue *SlCYP90A5* has similar expression profile. CBF/DREB1 transcription factor plays role in cold response [64] and is over-represented in *SlCYP72A184*, *SlCYP85A1* and *SlCYP96A48* genes. These genes can be candidate for cold stress tolerance in tomato. Intron map along with their phases and gain/loss events plays a crucial role in understanding the evolution of gene families within phylogenetic group. Conserved introns are ancient elements and present with similar intron phase [65]. Intron phase changes due to intron sliding events or changing intron-exon boundaries with one or two nucleotides [66]. Introns tend to maintain their phases during evolution, given that changes in intron phases occur rarely. In the mulberry P450, maximum genes contain one and two introns that were comparable with tomato P450 introns [15]. Both conserved introns were evolved in clan71 gene families due to gene duplication events. In *Arabidopsis,* two conserved introns were absent from non-A type P450 gene families whereas they appeared in A-type P450 gene families [31]. It is observed that conserved intron I13 evolved gradually and conserved intron I14 lost from *Sl*P450 genes during the course of evolution. Intron gain was observed in the A-type of *Sl*P450 genes which was absent in the ancestral (Non-A) gene families. Hence, this data support the intron late view of intron evolution [30, 31].

The expression evidence to the genes profoundly depends on developmental stages, age of the plant, environmental conditions, extent of expression, tissue specificity and biotic or abiotic stress. In the present study, only 31.33% P450 genes showed evidence of expression which could be compared with rice (49.81%) [67] and soybean (31.92%) [14]. In mulberry, Ma et.al. (2014) have identified 173 P450 genes which were further divided into five clusters for expression profile and found that the maximum 23.6% P450 genes were expressed [15]. Present study is conducted on the available RNA sequencing data of different tissues of tomato. The data was not obtained by challenging plant with any pathogen or exposing plants to the different stress conditions. Following possibilities can be asserted in the given case: i) remaining genes have developmental specificity or ii) it is expressed in different biotic or abiotic stress conditions or iii) it is present in the in-detectable level or iv) is inactive. The digital expression analysis provides global landscape could be instrumental to study various tissue specific P450. The promoter analysis suggested *Sl*P450 promoter motifs are driving tissue specific expression. Thus present study may enable researchers to select appropriate candidate gene from huge repertoire of *Sl*P450 for detailed functional characterization.

## Conclusion

The Tomato genome has a greater number of P450 clans as compared to *Arabidopsis* with variable number of P450 genes in each clan. Phylogenetic tree analysis provided the information about the functional evolution of P450 gene family in tomato. In intron map, gain and loss of conserved introns reveals P450 gene family evolution in tomato plant. Digital and experimental expression profile suggests tissues specific highly expressed P450 genes that could be potential candidates for further study. The promoter motifs driving the higher expression of P450 in analysed tissues types can be further evaluated using functional genomics for traits of economic importance. Thus, this study provides solid foundation for functional characterization of candidate genes with their biological significance.

## Additional files

Additional file 1: Table of *Sl*P450 summary file 233 *Sl*P450 sequences (genomic, transcript, CDS and protein) are provided with the universal names, Sol genomics id and phylogenetic groups. The NCBI accession numbers of Arabidopsis, Poplar and Potato P450 protein sequences used in phylogeny are listed. The sheet 2 represents percent identity matrix of all the *Sl*P450 proteins. (XLSX 197 kb)

Additional file 2: Table of primer sequences used in experimentation. Primer sequences of genes used RT-qPCR analysis. (XLSX 8 kb)

Additional file 3: *Sl*P450 phylogenetic tree inferred using Maximum likelihood method. Phylogenetic tree is constructed by applying maximum likelihood method with 1000 ultrafast bootstrap replicates using LG + F + I + G4 as best-fit substitution model. (TIF 13827 kb)

Additional file 4: Intron analysis summary. Data for Intron map constructions and distribution. (XLSX 34 kb)

Additional file 5: Promoter analysis Table. Promoter analysis of all the protein coding genes in tomato with promoter motifs count. (XLSX 81994 kb)

Additional file 6: Tissue specific expression of P450 with FPKM count. Highly expressed tomato P450 genes from heat map with their FPKM count. (XLSX 11 kb)

Additional file 7: Table of P450 orthologue gene count in related plants. Comparison of tomato P450 gene along with other related plants P450 orthologue gene count. (DOCX 20 kb)

## Abbreviations

*At*P450: *Arabidopsis thaliana* P450
BLAST: Basic Local Alignment Search Tool
CYP: Cytochrome P450
FPKM: Fragments Per Kilobase of Transcript per Million mapped reads
MEGA: Molecular Evolutionary Genetics Analysis
P450: Cytochrome P450
RT-qPCR: Real Time Quantitative Polymerase Chain Reaction
*Sl*P450: *Solanum lycoperscium* Cytochrome P450
