# Supplementary material for: Phylogenomic analysis of cytochrome P450 multigene family and their differential expression analysis in Solanum lycopersicum L. suggested tissue specific promoters
Source: BMC Genomics. 2019 Feb 7;20:116. doi: 10.1186/s12864-019-5483-x (PMC6367802; doi:10.1186/s12864-019-5483-x)
Supplement: Supplementary file 7 — Table of P450 orthologue gene count in related plants. Comparison of tomato P450 gene along with other related plants P450 orthologue gene count. (DOCX 20 kb) [file 12864_2019_5483_MOESM7_ESM.docx]

Comparison of CYP450 from different plants:

**A-type of Cytochrome P450**:

|  | Soybean | Arabidopsis | Flax | Poplar | Moss | Grape | Medicago | Rice | tomato |
| --- | --- | --- | --- | --- | --- | --- | --- | --- | --- |
| Clan71 |  |  |  |  |  |  |  |  |  |
| CYP71 | 55 | 52 | 48 | 25 | 0 | 24 | 37 | 84 | 43 |
| CYP73 | 3 | 1 | 6 | 3 | 4 | 3 | 1 | 3 | 1 |
| CYP75 | 7 | 1 | 6 | 3 | 0 | 11 | 0 | 3 | 2 |
| CYP76 | 14 | 8 | 26 | 13 | 0 | 24 | 6 | 29 | 18 |
| CYP77 | 4 | 5 | 7 | 3 | 0 | 2 | 2 | 2 | 3 |
| CYP78 | 11 | 6 | 9 | 10 | 3 | 7 | 1 | 8 | 5 |
| CYP79 | 5 | 7 | 9 | 4 | 0 | 9 | 3 | 4 | 5 |
| CYP80 | 0 | 0 | 0 | 6 | 0 | 6 | 0 | 0 | 5 |
| CYP81 | 12 | 18 | 13 | 28 | 0 | 21 | 5 | 12 | 10 |
| CYP82 | 24 | 5 | 18 | 10 | 0 | 34 | 10 | 0 | 9 |
| CYP83 | 12 | 1 | 0 | 5 | 0 | 0 | 9 | 0 | 0 |
| CYP84 | 3 | 2 | 10 | 3 | 0 | 3 | 3 | 3 | 1 |
| CYP89 | 8 | 7 | 7 | 10 | 0 | 14 | 9 | 14 | 2 |
| CYP92 | 2 | 0 | 0 | 8 | 0 | 6 | 1 | 9 | 7 |
| CYP93 | 13 | 1 | 5 | 4 | 0 | 4 | 8 | 3 | 1 |
| CYP98 | 2 | 3 | 4 | 5 | 1 | 1 | 1 | 2 | 3 |
| CYP99 | 0 | 0 | 0 | 0 | 0 | 0 | 0 | 2 | 0 |
| CYP701 | 2 | 1 | 5 | 1 | 1 | 1 | 1 | 5 | 1 |
| CYP703 | 1 | 1 | 1 | 1 | 3 | 1 | 0 | 1 | 0 |
| CYP705 | 0 | 26 | 0 | 0 | 0 | 0 | 1 | 0 | 11 |
| CYP706 | 3 | 7 | 15 | 5 | 0 | 9 | 1 | 4 | 1 |
| CYP723 | 0 | 0 | 0 | 0 | 0 | 0 | 0 | 2 | 0 |
| CYP712 | 2 | 2 | 1 | 9 | 0 | 2 | 1 | 0 | 1 |
| CYP736 | 12 | 0 | 0 | 6 | 0 | 8 | 1 | 0 | 8 |
| Other | 0 | 0 | 0 | 0 | 29 | 0 | 0 | 0 | 0 |
| Total | 195 | 154 | 190 | 162 | 41 | 190 | 101 | 190 | 137 |

**Non A type of Cytochrome P450**:

|  | Soybean | Arabidopsis | Flax | Poplar | Moss | Grape | Medicago | Rice | tomato |
| --- | --- | --- | --- | --- | --- | --- | --- | --- | --- |
| CLAN51 |  |  |  |  |  |  |  |  |  |
| CYP51 | 2 | 1 | 2 | 2 | 1 | 2 | 1 | 10 | 1 |
| CYP72 clan |  |  |  |  |  |  |  |  |  |
| CYP72 | 12 | 9 | 4 | 6 | 0 | 22 | 7 | 13 | 20 |
| CYP709 | 0 | 3 | 21 | 1 | 0 | 1 | 1 | 9 | 0 |
| CYP714 | 6 | 2 | 1 | 6 | 0 | 6 | 3 | 5 | 3 |
| CYP715 | 6 | 1 | 2 | 2 | 0 | 1 | 1 | 1 | 1 |
| CYP721 | 2 | 1 | 3 | 6 | 0 | 5 | 1 | 2 | 1 |
| CYP734 | 3 | 1 | 2 | 2 | 0 | 2 | 1 | 4 | 2 |
| CYP735 | 3 | 1 | 2 | 2 | 0 | 1 | 1 | 2 | 1 |
| CYP749 | 0 | 0 | 0 | 9 | 0 | 0 | 0 | 0 | 2 |
| CYP74 clan |  |  |  |  |  |  |  |  |  |
| CYP74 | 6 | 2 | 10 | 6 | 3 | 7 | 4 | 4 | 7 |
| CYP85 clan |  |  |  |  |  |  |  |  |  |
| CYP85 | 5 | 2 | 4 | 3 | 0 | 2 | 1 | 1 | 2 |
| CYP87 | 2 | 1 | 5 | 12 | 0 | 7 | 2 | 11 | 3 |
| CYP88 | 3 | 2 | 5 | 2 | 0 | 2 | 3 | 1 | 3 |
| CYP90 | 12 | 4 | 8 | 7 | 0 | 4 | 4 | 5 | 4 |
| CYP702 | 0 | 6 | 0 | 0 | 0 | 0 | 0 | 0 | 0 |
| CYP707 | 10 | 4 | 10 | 7 | 0 | 5 | 3 | 3 | 4 |
| CYP708 | 0 | 4 | 0 | 0 | 0 | 0 | 0 | 0 | 0 |
| CYP716 | 7 | 2 | 1 | 17 | 1 | 15 | 3 | 0 | 6 |
| CYP718 | 1 | 1 | 1 | 1 | 1 | 15 | 0 | 0 | 1 |
| CYP720 | 2 | 1 | 2 | 1 | 0 | 1 | 1 | 0 | 1 |
| CYP722 | 2 | 1 | 5 | 1 | 0 | 1 | 1 | 1 | 2 |
| CYP724 | 1 | 1 | 4 | 2 | 0 | 2 | 0 | 1 | 2 |
| CYP728 | 2 | 0 | 0 | 7 | 0 | 6 | 0 | 11 | 1 |
| CYP729 | 0 | 0 | 0 | 1 | 0 | 0 | 1 | 2 | 0 |
| CYP733 | 3 | 0 | 0 | 1 | 0 | 1 | 0 | 1 | 1 |
| CYP86 clan |  |  |  |  |  |  |  |  |  |
| CYP86 | 9 | 11 | 12 | 8 | 2 | 6 | 3 | 5 | 4 |
| CYP94 | 14 | 6 | 9 | 13 | 2 | 9 | 4 | 18 | 8 |
| CYP96 | 7 | 13 | 14 | 9 | 0 | 5 | 5 | 12 | 5 |
| CYP704 | 5 | 3 | 7 | 6 | 6 | 6 | 14 | 7 | 7 |
| CYP97 clan |  |  |  |  |  |  |  |  |  |
| CYP97 | 5 | 3 | 5 | 3 | 3 | 3 | 4 | 3 | 2 |
| CYP710 clan |  |  |  |  |  |  |  |  |  |
| CYP710 | 2 | 4 | 1 | 1 | 2 | 1 | 1 | 4 | 1 |
| CYP711 clan |  |  |  |  |  |  |  |  |  |
| CYP711 | 4 | 1 | 2 | 2 | 0 | 1 | 2 | 5 | 1 |
| CYP727 clan |  |  |  |  |  |  |  |  |  |
| CYP727 | 1 | 0 | 2 | 2 | 0 | 0 | 0 | 1 | 0 |
| CYP746 clan |  |  |  |  |  |  |  |  |  |
| CYP746 | 0 | 0 | 0 | 0 | 1 | 0 | 0 | 0 | 0 |
| Other | 0 | 0 | 0 | 0 | 9 | 0 | 0 | 0 | 0 |
| Total | 137 | 91 | 144 | 148 | 31 | 139 | 72 | 142 | 96 |
